# L-Rhamnose Dehydrogenase LraA of *Aspergillus niger* Shows High Substrate Specificity Matching Its Expression Profile

**DOI:** 10.3390/jof11040301

**Published:** 2025-04-10

**Authors:** Agata Terebieniec, Li Xu, Mao Peng, Miia R. Mäkelä, Ronald P. de Vries

**Affiliations:** 1 Fungal Physiology Group, Westerdijk Fungal Biodiversity Institute, Uppsalalaan 8, 3584 CT Utrecht, The Netherlands; terebieniecagata@gmail.com (A.T.); xuli_lixu1125@163.com (L.X.); m.peng@wi.knaw.nl (M.P.); 2 Department of Bioproducts and Biosystems, Aalto University, P.O. Box 16100, FI-02150 Espoo, Finland; miia.makela@aalto.fi

**Keywords:** L-rhamnose pathway, L-rhamnose dehydrogenase, substrate specificity, expression profile

## Abstract

L-rhamnose is one of the main monomeric sugars of rhamnogalacturonan I and II, which are polysaccharide components of pectin. In the ascomycete fungus *Aspergillus niger* it is metabolized through the non-phosphorylated L-rhamnose pathway, of which the first step is catalyzed by L-rhamnose dehydrogenase (LraA), converting L-rhamnose into L-rhamnono-γ-lactone. This enzyme belongs to PFAM PF00106, unlike most of other reductases/dehydrogenases involved in fungal sugar catabolism that are typically assigned to PF00248 and PF00107. The enzymes of those families have broad substrate specificity and in some cases have been shown to be involved in multiple pathways. In this study we heterologously produced and biochemically characterized *A. niger* LraA and studied its expression on a set of monosaccharides. This revealed that, in contrast to other metabolic redox enzymes, LraA is highly specific for L-rhamnose and has no activity on most other substrates tested in this study. This specificity is matched by a highly specific expression profile, which only shows significant expression on L-rhamnose. It therefore can be concluded that LraA has evolved with a highly specific function in fungal sugar catabolism, unlike most other sugar reductases/dehydrogenases described so far. The high specificity of LraA also affects its biotechnological applications, as it may benefit L-rhamnose-based processes, but would be less suitable for applications involving conversion of multiple sugars.

## 1. Introduction

**Figure 1:**
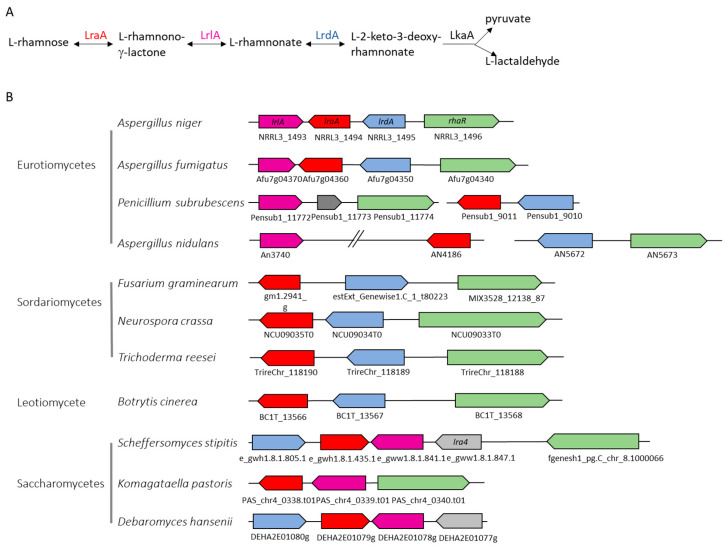
The fungal non-phosphorylated L-rhamnose metabolic pathway (**A**) and the LRA gene cluster in fungi (**B**). Red = *lraA* and its orthologs; purple = *lrlA* and its orthologs; blue = *lrdA* and its orthologs; green = *rhaR* transcriptional regulator and its orthologs; grey = genes not belonging to the cluster. Gene numbers below the graphs are obtained from JGI MycoCosm (https://mycocosm.jgi.doe.gov/mycocosm/home, accessed on 15 January 2025) (Table S1).

Plant cell walls are mainly composed of homo- and heteropolymers, with pectin being the most complex polysaccharide. Pectin contains four structural elements: homogalacturonan (HG), xylogalacturonan (XG), rhamnogalacturonan I (RG-I), and rhamnogalacturonan II (RG-II) [1,2,3,4]. L-Rhamnose is one of the main components of pectin, and many microorganisms are able to transport this sugar into their cells and metabolize it in the absence of a more preferable carbon source [5,6,7,8]. Bacteria use a phosphorylated pathway that converts L-rhamnose to dihydroxyacetone phosphate (DHAP) [9,10,11]. The non-phosphorylated L-rhamnose metabolic pathway is characteristic for fungi [5,12,13] (Figure 1) but has also been identified in three bacterial species, *Azotobacter vinelandii*, *Burkholderia cenocepacia*, and *Sphingomonas* sp. [13,14]. It was initially described in *Aureobasidium pullulans* (formerly *Pullularia pullulans*) [15] but has also been identified in other fungi [5,12,13,16,17]. In this pathway, L-rhamnose is converted into pyruvate and lactaldehyde through a cascade of four reactions catalyzed by L-rhamnose-1-dehydrogenase (LRA1/LraA), L-rhamnono-γ-lactonase (LRA2/LrlA), L-rhamnonate dehydratase (LRA3/LrdA), and L-2-keto-3-deoxyrhamnonate aldolase (LRA4/LkaA) (Figure 1A) [5,13,16,18,19]. In most fungi, these enzymes are encoded in the LRA gene cluster, although the organization of the cluster varies between species (Figure 1B) [5,13,16]. The fourth gene of the pathway (LRA4/LkaA) is not present in the cluster in *Aspergillus niger* and most other fungi [5,18].

Sugar reductases and dehydrogenases are an essential part of *A. niger* carbon metabolism [20]. Studies on their characteristics and their involvement in different metabolic pathways and analysis of their phylogenetic relationship are important to obtain a deeper understanding of the complexity of the carbon metabolism as well as the role of *A. niger* in plant biomass degradation. Based on recent studies, these fungal enzymes often have broad substrate specificity and can even be involved in multiple pathways [21]. However, a previous genetic study on the L-rhamnose pathway suggested high specificity of the involved enzymes [18]. So far, no detailed biochemical analysis of fungal L-rhamnose dehydrogenases has been reported, nor has this been related to its in vivo functionality through expression profiling of the corresponding gene. Therefore, in this study we performed a detailed analysis of L-rhamnose dehydrogenase (LraA) from *A. niger*, with respect to substrate specificity and gene expression profile, and demonstrated that it is specifically involved in L-rhamnose catabolism. This emphasizes its applications in biotechnology and metabolic engineering as it will specifically convert L-rhamnose but is unlikely to affect other metabolic pathways.

## 2. Materials and Methods

### 2.1. Phylogenetic Analysis

Amino acid sequences of proteins belonging to PFAM family PF00106 were retrieved from JGI MycoCosm. NRLL3_1494 (LraA) from *A. niger* was used as a query for BlastP search using standard settings in *Aspergillus nidulans*, *Chaetomium globosum*, *Neurospora crassa*, *Penicillium subrubescens*, *Podospora anserina*, and *Trichoderma reesei* genomes. NRRL3_10884 (L-xylulose reductase, LxrA) from *A. niger* [22] and its ortholog in *A. nidulans*, AN10169, were used as an outgroup. The sources of the genomic data are listed in Supplementary Table S1. Sequences were aligned using MAFFT [23] and manually corrected. The phylogenetic tree was constructed using MEGA6 [24] with the Maximum Likelihood algorithm, and the Poisson correction model, using 500 bootstraps. The representative Maximum Likelihood tree was then displayed with bootstrap values at the nodes if the support was at least 50%.

### 2.2. Transcriptome Analysis

Previously published RNAseq data [20,25] were used in this study to analyze the expression of *A. niger* L-rhamnose-related genes in more detail. All species were pre-grown in complete medium containing 1% D-fructose for 16 h and transferred to 50 mL minimal medium supplemented with 25 mM D-glucose, D-fructose, D-xylose, L-arabinose, D-mannose, D-galactose, L-rhamnose, or D-galacturonic acid. The mycelium was harvested after 2 h of incubation, dried between tissue paper, and frozen in liquid nitrogen. Total RNA was isolated from the mycelium and used for transcriptome analysis as described previously [20]. Gene expression values of L-rhamnose-utilizing genes were visualized with the ‘ComplexHeatmap’ R package [26]. Statistical analysis was performed using DESeq2 [27].

The reads from each of the transcriptome sequencing (RNA-seq) samples were deposited in the Sequence Read Archive at NCBI under the following accession numbers: *A. niger* SRP448993, SRP449003–SRP449007, SRP449023, SRP449039, SRP449049, SRP449062, SRP449079–SRP449081, SRP449083–SRP449085, SRP449089, SRP449068–SRP449070, SRP449098, SRP449125, SRP449138, SRP449141, SRP449142, SRP449151, and SRP449193; *A. nidulans* SRP262827–SRP262853; *P. subrubescens* SRP246823–SRP246849; *T. reesei* SRP378720–SRP378745.

### 2.3. Growth Profile Analysis

*A. niger* N402, *A. nidulans* FGSC A4, *P. subrubescens* CBS 132785, and *T. reesei* QM6a used in this study were grown at 30 °C using minimal medium (MM, pH 6) or complete medium (CM, pH 6) [20] with the appropriate carbon source. For solid cultivation, 1.5% (*w*/*v*) agar was added in the medium. Spores were harvested from complete medium (CM) agar plates in N-(2-acetamido)-2-aminoethanesulfonic acid (ACES) buffer (10 mM N-(2-acetamido)-2-amino-ethanesulfonic acid, 0.02% Tween 80, pH 6.8), after five days of growth, and counted using a hemocytometer. Growth profiling plates were inoculated with 1000 spores in 2 μL ACES buffer and incubated at 30 °C for 5 days.

### 2.4. Construction of an Expression Plasmid

The cDNA of L-rhamnose dehydrogenase (*lraA*, NRRL3_1494) was codon-optimized and synthesized into pET28a(+) plasmid for production in *Escherichia coli* (GenScript Biotech, Leiden, the Netherlands). The pET28a(+) containing *lraA* was transformed into *E. coli* DH5α for propagation. Then, the plasmid was extracted and transformed into *E. coli* Arctic Express (Novagen, Merck, Darmstadt, Germany) according to the manufacturer’s recommendation. The transformants were selected on Luria Bertani (LB) medium supplemented with 25 mg × L^−1^ kanamycin and 20 mg × L^−1^ gentamycin. Positive colonies were verified by colony PCR using T7 promoter and terminator-specific primers (T7 promoter = 5′-TAA TAC GAC TCA CTA TAG GG-3′; T7 terminator = 5′-GCT AGT TAT TGC TCA GCG G-3′).

### 2.5. Recombinant Protein Production and Purification

Transformed *E. coli* was grown to an OD600 of 0.8 in LB medium containing 20 mg × L^−1^ gentamycin and 25 mg × L^−1^ kanamycin at 37 °C in the rotary shaker (250 rpm). The production of LraA was induced by addition of isopropyl β-D-1-thiogalactopyranoside (IPTG) to a final concentration of 0.1 mM and incubated overnight (16–18 h) at 10 °C with shaking (250 rpm). The cells were harvested by centrifugation at 8000× *g* for 15 min at 4 °C and the cell pellet was resuspended in 30 mL of BugBuster Protein Extraction Reagent (Novagen, Merck, Darmstadt, Germany) containing 2 µL Benzonase Nuclease (10,000 U) (Merck Millipore, Darmstadt, Germany). After 20 min incubation at 4 °C with rotating mixing, the cell debris was removed by centrifugation at 8000× *g* for 20 min at 4 °C. Supernatants were filtered (45 µm, Whatman, GE Healthcare Life Sciences, Pittsburgh, PA, USA) and applied to an ÄKTA start chromatography system (Cytiva Life Sciences, Marlborough, MA, USA) equipped with 1 mL HisTrap FF column (Cytiva Life Sciences, Marlborough, MA, USA) that was equilibrated with 20 mM HEPES, 20 mM imidazole, and 400 mM NaCl, pH 7.5. The protein was eluted with 10 mL of 20 mM HEPES, 400 mM imidazole, and 400 mM NaCl, pH 7.5, at a flow rate of 1.0 mL × min^−1^. Collected fractions containing the enzyme were pooled and verified by SDS–PAGE. The theoretical molecular mass of the protein was calculated based on its amino acid sequence using ExPASy (https://web.expasy.org/compute_pi/, accessed on 15 October 2024) [28]. The LraA solution was desalted and concentrated with 20 mM HEPES, pH 7.0, using a Sartorius Vivaspin 20 centrifugal concentrator (10,000 Da PES membrane, Sartorius, Göttingen, Germany). All purification steps were performed at 4 °C. Concentration of the purified protein was determined using the Pierce BCA Protein Assay Kit (Thermo Fisher Scientific, Waltham, MA, USA).

### 2.6. Enzyme Assays

The enzyme activity was measured in a reaction mixture containing 100 mM Tris-HCl buffer (pH 8.0), 1 mM nicotinamide adenine dinucleotide (NAD+), 10 mM of substrate, and 9.5 μg × mL^−1^ of purified enzyme at 25 °C. The sugar compounds used for substrate specificity analysis were L-rhamnose, L-fucose, L-lyxose, D-glucose, D-fructose, D-arabinose, D-xylose, D-mannose, D-galactose, L-arabinose, D-ribose, D-erythrose, and L-tagatose. The formation of nicotinamide adenine dinucleotide + hydrogen (NADH) was followed by measuring the absorbance at 340 nm (extinction coefficient = 6.22 × 10^−3^ M^−1^ cm^−1^) in flat-bottom microtiter plates (Grainer Bio-One, Kremsmünster, Austria) in a microplate reader (FLUOstar OPTIMA, BMG LABTECH, Ortenberg, Germany). Cofactor specificity was analyzed by using 1 mM nicotinamide adenine dinucleotide phosphate (NADP+) instead of NAD+. The kinetic constants were calculated from the Michaelis Menten equation fitted to the measured data.

## 3. Results and Discussion

### 3.1. The lraA Gene Is Expressed Exclusively on L-Rhamnose Unlike Its Close Homolog of PF00106

**Figure 2:**
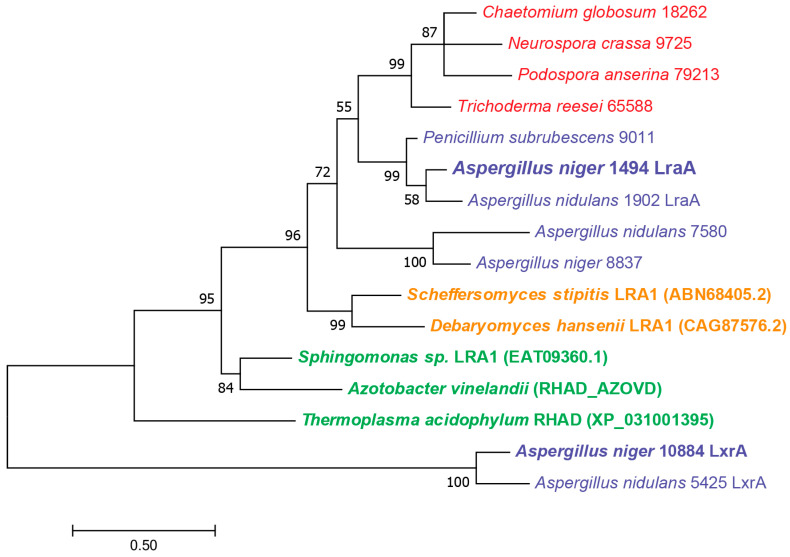
Phylogenetic analysis of LraA and its homologs in selected fungi and bacteria. Maximum Likelihood tree (500 bootstraps) is a representative of an MAFFT amino acid alignment. Sequences in bold represent characterized enzymes. The numbers behind the species are protein IDs from JGI MycoCosm (https://mycocosm.jgi.doe.gov/mycocosm/home, accessed on 17 February 2025). The numbers in brackets are NCBI accession numbers. Blue, red, orange, and green fonts indicate Eurotiomycete enzymes, Sordariomycete enzymes, characterized yeast enzymes [13,19], and characterized bacterial enzymes [14,29,30], respectively. *A. niger* LxrA [25] and its ortholog from *A. nidulans* were used as an outgroup.

In a previous study, detailed analysis of the pentose catabolic pathway (PCP) in *A. niger* revealed the involvement of several reductases that had significant sequence homology [21]. To evaluate whether *A. niger* also contains additional LRA-encoding genes, we performed a BlastP search that revealed only one possible candidate, NRRL3_8837, with 56% identity (Figure S1). A BlastP search using *A. niger* LraA on the genomes of selected fungi showed that each of those species have an LraA ortholog. Phylogenetic analysis demonstrated that *A. nidulans* AN1902 and *P. subrubescens* 9011 are most closely related to LraA (Figure 2), which matches the taxonomic distance of these species. The sister clade contains LraA orthologs from Sordariomycetes. Interestingly, the only LraA paralog (NRRL3_8837) only has an ortholog in *A. nidulans* (AN7580), and these proteins are more distant from LraA than proteins from the Sordariomycetes clade. All bootstrap values are over 50, and most are over 90, indicating high support for the whole tree. As this also matches their taxonomic relationships, the tree can be considered to reliably present the relationships between these enzymes. The placement of the paralogous group of *Aspergillus* enzymes between LraA and the bacterial LRAs makes them interesting candidates for future studies, especially considering that *A. niger* 8837 is not specifically expressed on L-rhamnose (Figure S2). Its unspecific expression profile does not provide direct clues to its possible function, which means that heterologous expression and in vitro enzyme assays with a set of sugars may be the best approach for studying this.

**Figure 3:**
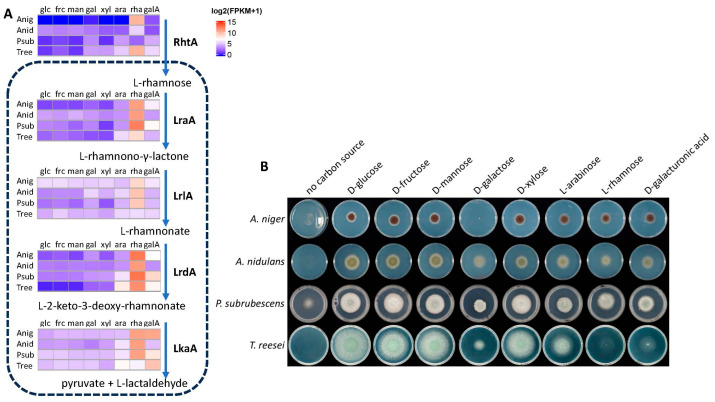
Expression (**A**) and growth (**B**) profiles of *A. niger*, *A. nidulans*, *P. subrubescens*, and *T. reesei*. (**A**) Expression of genes involved in L-rhamnose transport and catabolism. Transcriptome data were obtained from [20,25]. Anig = *A. niger*, Anid = *A. nidulans*, Psub = *P. subrubescens*, Tree = *T. reesei*, glc = D-glucose, frc = D-fructose, man = D-mannose, xyl = D-xylose, ara = L-arabinose, rha = L-rhamnose, gala = D-galacturonic acid. (**B**) Growth profiles of *A. niger*, *A. nidulans*, *P. subrubescens*, and *T. reesei* on several monosaccharides and D-galacturonic acid.

To evaluate whether NRRL3_8837 could be a second L-rhamnose dehydrogenase-encoding gene, we analyzed its expression in transcriptomic data from *A. niger* on eight monosaccharides [20,25] and compared this to the expression of *lraA*. While *lraA* is specifically expressed at high levels on L-rhamnose (Figure 3, Supplementary Table S2), NRRL3_8837 is poorly expressed on L-rhamnose (Figure S2) and therefore is unlikely to have a function as a second L-rhamnose dehydrogenase in *A. niger* L-rhamnose catabolism. This is also supported by the lack of growth of the *A. niger lraA* deletion mutant on L-rhamnose [18]. Additionally, analysis of the deletion of *lraA* (AN4186) in *A. nidulans* also resulted in nearly fully impaired growth on L-rhamnose, and no L-rhamnose dehydrogenase activity was detected in the cell-free extract of this mutant [12]. Taken together, there is no evidence for a functional paralog of LraA in *A. niger*.

### 3.2. Expression of L-Rhamnose Catabolic Pathway Genes in Four Fungi Correlates with Their Growth on L-Rhamnose

To obtain a deeper understanding of L-rhamnose catabolism in *A. niger* and other fungi we performed an expression analysis of all genes involved in this pathway as well as their orthologs in three other fungi. These fungi were chosen as we have identical expression data available for them, generated under the same conditions and using the same methodologies, which minimizes the variation due to the experimental approach. These three fungi were previously chosen as *A. nidulans*, which is a relative of *A. niger* and a broadly used model species for fungal biology, while the other two are biotechnologically relevant fungi that are from close (*P. subrubescens*) or more distant (*T. reesei*) fungal genera. In addition, we included the expression of the previously identified L-rhamnose transporter-encoding gene RhtA from *A. niger* [7]) and its orthologs, and we correlated these results with growth profile analysis (Figure 3).

The Eurotiomycete species show similar growth on most of the studied monosaccharides. The only exception is the lack of growth of *A. niger* on D-galactose which has been shown to be due to non-functional D-galactose transport during germination [31]. The Sordariomycete *T. reesei* shows poor growth on D-galacturonic acid, while growth on L-rhamnose is almost completely abolished. A possible explanation for this could be the low expression of the *lrlA* and *lkaA* orthologs in *T. reesei*, which is significantly lower than those in most of the other species (Figure 3). As the *T. reesei* orthologs from *lrlA* and *lkaA* are not present in the cluster, other genes may encode these functions in *T. reesei.* However, the poor growth on L-rhamnose matches better with a poor expression of pathway genes.

Interestingly, the *rhtA* orthologs of *A. nidulans* and *P. subrubescens* show very low expression on L-rhamnose (Figure 3), putting their involvement in L-rhamnose uptake in question. In a recent evaluation of sugar transporters of these species [25], several other candidate L-rhamnose transporters were identified, which may explain the growth of these two species despite low *rhtA* expression.

### 3.3. LraA Is Highly Specific for L-Rhamnose

**Figure 4:**
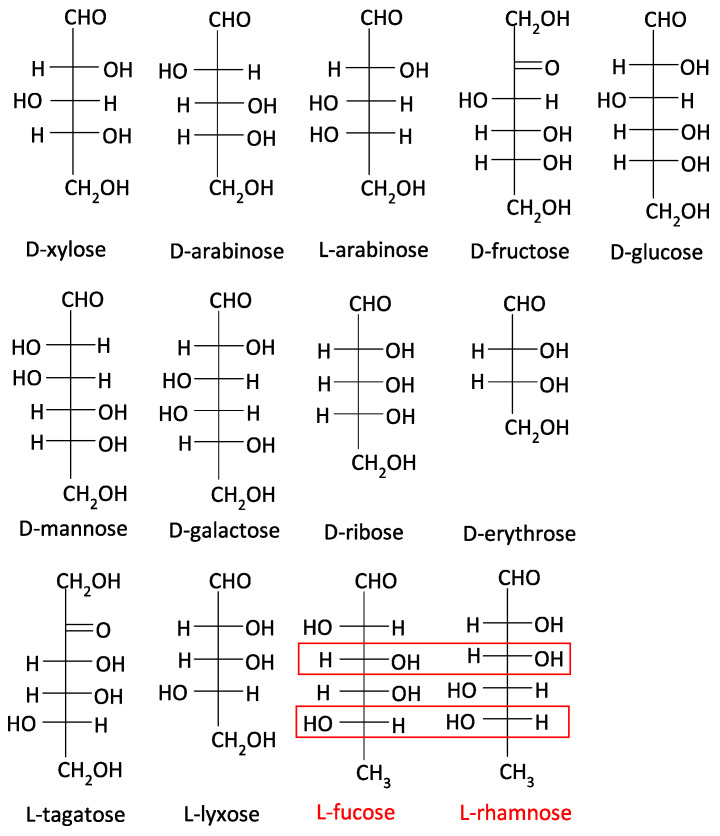
Fisher projections of substrates used in LraA substrate specificity analysis. The structures present the chiral configurations of the different sugars and demonstrate the high specificity of LraA. The red boxes indicate identical chiral centers for L-fucose and L-rhamnose.

To determine the substrate specificity of *A. niger* LraA, we recombinantly produced it in *E. coli*. The theoretical molecular mass of this enzyme is 28.7 kDa, which matches the value observed on SDS–PAGE after production of the enzyme (Figure S3). The substrate specificity of LraA was tested with 13 substrates (Figure 4), of which two were ketoses and 11 aldoses. LraA is only highly active on L-rhamnose (63.4 ± 5.7 U·mg^−1^), with a 55-fold lower activity on L-fucose (1.15 ± 0.43 U·mg^−1^). No activity was observed on any of the other substrates. These two sugars share the same configuration on two chiral centers: (R) on C-3 and (S) on C-5 (Figure 4), suggesting that this may be required for LraA to be active.

**Figure 5:**
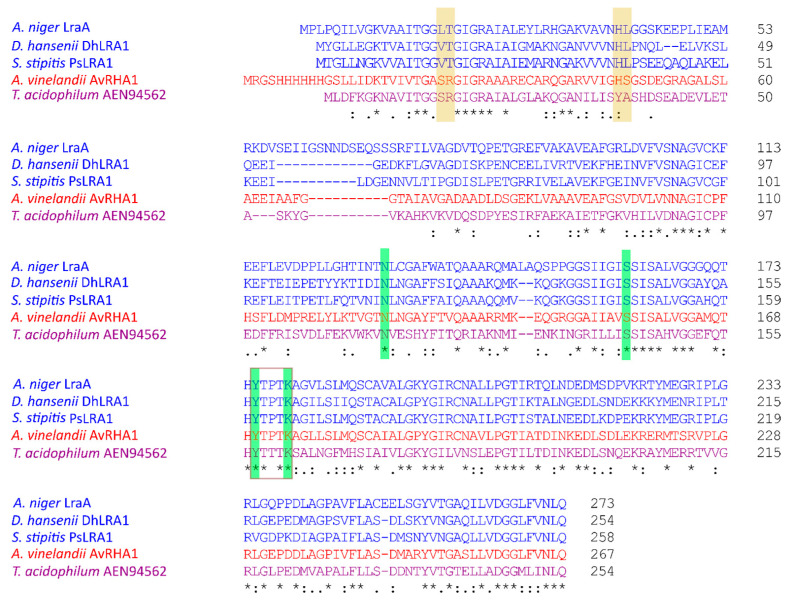
Clustal Omega alignment of characterized L-rhamnose dehydrogenases. Blue font = fungal, red font = bacterial, and purple font = archaeal L-rhamnose dehydrogenases. Yellow highlight = conserved Thr or Arg in cofactor-binding motif; green highlight = catalytic tetrad; brown frame = catalytic site. Symbols underneath the alignment mean: * = fully conserved amino acid; : and . similar type of amino acid.

As the activity was highly specific for L-rhamnose, and the activity on L-fucose was very low, which would make kinetic values for L-fucose less convincing, we only calculated kinetic constants for the conversion of L-rhamnose. The results of this analysis were compared to the values of previously published microbial L-rhamnose dehydrogenases and are shown in Table 1. LraA has a high affinity for L-rhamnose which is comparable to the bacterial enzyme AvLRA1 [13], which also has a similar turnover as LraA. LraA has a strict dependency on NAD+ as a cofactor, which confirms studies with L-rhamnose dehydrogenases from the yeasts *S. stipitis* and *D. hansenii* [13,19,32]. Interestingly, while all characterized fungal L-rhamnose dehydrogenases are only active with NAD+ [13,19,32], bacterial L-rhamnose dehydrogenases are able to use both NAD+ and NADP+, and archaeal enzymes display NADP+ cofactor specificity [29,30]. To identify possible reasons for this, we performed a sequence alignment of five characterized microbial L-rhamnose dehydrogenases using Clustal Omega version 1.2.2 [33], which showed the differences in their N-terminal cofactor-binding motifs (Figure 5). Threonine (T) residue Thr-19 in *A. niger* LraA is conserved in other fungal L-rhamnose dehydrogenases and indicates NAD+ dependency, while arginine (R) at the corresponding position designates NADP+ preference. Arginine favors the binding of NADP+ due to the presence of a negatively charged phosphate in this cofactor [34,35]. The residues for cofactor-binding were studied in more detail in a bacterial LRA [30]. An R15T mutant of this enzyme modified specificity to NAD^+^, which is consistent with the presence of a T at this position in *A. niger* LraA (Figure 5). Of the other three residues implicated in coenzyme specificity in this study [30], *A. niger* LraA contains an L at the position of S14 and S37 of the bacterial enzyme, while H36 is conserved (Figure 5). The S to L mutations do not seem to alter the cofactor preference to NADP^+^, as *A. niger* LraA is strictly NAD^+^-dependent. The substrate-binding sites identified in the bacterial enzyme [30] are all conserved in *A. niger* LraA, but the bacterial enzyme has a broader substrate specificity than *A. niger* LraA, suggesting the involvement of additional residues that limit its substrate range.

L-rhamnose dehydrogenases belong to the large and functionally heterogeneous short-chain dehydrogenases/reductases superfamily (SDR) [36]. *A. niger* LraA contains conserved motifs for SDR, such as the previously mentioned N-terminal glycine-rich motif TGGLTGIGR (residues 15–23 in LraA), which is a nucleotide-binding Rossmann fold, and a catalytic tetrad (N131-S161-Y175-K179) with Y and K located in the catalytic center and responsible for substrate-binding [37]. All aligned enzymes (Figure 5) belong to the SDR superfamily, and they share the identical catalytic tetrad, which suggests the same mechanism of catalysis. Interestingly, *A. niger* LraA is more specific than the other L-rhamnose dehydrogenases in that it was only active on L-rhamnose and L-fucose. PsLRA1 from *S. stipitis*, DhLRA1 from *D. hansenii*, and AvLRA1 from the bacterium *A. vinelandii* showed a broader substrate specificity with activity on L-rhamnose, L-lyxose, L-fucose, and L-mannose [17,30], while Rha1 from *S. stipitis* and the archaeal enzyme were active on L-rhamnose, L-lyxose, and L-mannose [19,29]. These four sugars share the same configuration on C-3, and L-rhamnose, L-fucose, and L-mannose also on C-5 (Figure 4). According to the low number of tested L-rhamnose dehydrogenases, it is difficult to build a strong conclusion on their substrate preferences. It seems, though, that for the substrate recognition by the fungal L-rhamnose dehydrogenases, the C-3 configuration is essential. An expansion in the number of characterized LRA-enzymes from a taxonomically diverse set of organisms in future studies would benefit the assignment of functional residues related to substrate preference.

The oxidation of a sugar to a sugar acid is not very common in Eukaryotes. Enzymes catalyzing those reactions act similarly, although they belong to different protein families. One example is the NAD-dependent dehydrogenase-catalyzed oxidation of D-galactose to D-galactono-lactone in the non-phosphorylated De Ley–Doudoroff pathway in *A. niger* [38]. This pathway is one of three described for D-galactose metabolism next to the Leloir and oxidoreductive pathways [39,40]. Another example is L-fucose metabolism, which is very similar to L-rhamnose catabolism. L-fucose is converted to L-fuconate, and finally, in the last step of the pathway, to pyruvate and L-lactaldehyde [15]. This may in part explain why these enzymes have higher substrate specificity than, e.g., the PF00248 sugar reductases, as mentioned before.

In conclusion, our results clearly indicate the lack of additional enzymes in *A. niger* that would be able to compensate for the loss of LraA in L-rhamnose catabolism. The *lraA* gene shows a highly specific expression pattern for L-rhamnose, and LraA has high biochemical specificity towards L-rhamnose, suggesting that this enzyme is specifically used in the L-rhamnose pathway. L-rhamnose is the least preferred sugar for *A. niger* as it is only consumed when no other sugars are present [41]. The low preference for this sugar may have prevented the need for the evolutionary development of redundancy of the enzymes of the L-rhamnose pathway. Although this low redundancy would cause a risk in a single mutation preventing use of L-rhamnose as a carbon source, this would not significantly impact growth of *A. niger* in natural environments. It can be a benefit for metabolic engineering in industrial applications as it provides a simpler approach to produce L-rhamnose-based metabolic intermediates as biochemicals. Future studies should address this and also the impact of introducing alternatives from other organisms that are less specific.

## Figures and Tables

**Table 1:** Kinetic constants of *A. niger* LraA and published LRA enzymes for L-rhamnose.

Organism	Enzyme	K_m_ [mM]	k_cat_ [min^−1^]	k_cat_/K_m_ [mM^−1^ min^−1^]	Reference
*Aspergillus niger*	LraA	2.4 ± 0.9	2149.2 ± 183.4	904.8 ± 30.7	This study
*Scheffersomyces stipitis*	PsLRA1	1.7 ± 0.0	1510.0 ± 20.0	885.0 ± 8.0	[13]
*S. stipitis*	Rha1	1.5 ± 0.0	NM	NM	[19]
*Debaryomyces hansenii*	DhLRA1	9.4 ± 1.1	2860.0 ± 230.0	307.0 ± 11.0	[13]
*Azotobacter vinelandii*	AvLRA1	2.2 ± 0.1	5010.0 ± 178.0	2250.0 ± 51.0	[13,30]
		2.6 ± 0.1 *	2230.0 ± 43.0 *	856.0 ± 28.0 *	
*Thermoplasma acidophilum*	-	0.5 *	1341.3 *	2915.9 *	[29]

* With NADP as a cofactor; NM: not mentioned; values are means ± SD, n = 3; SD for *T. acidophilum* was not mentioned in the original paper.

## Data Availability

The original contributions presented in this study are included in the article. Further inquiries can be directed to the corresponding author.

## Supplementary Materials

The following supporting information can be downloaded at https://www.mdpi.com/article/10.3390/jof11040301/s1: Figure S1: Alignment of *A. niger* LraA and 8837; Figure S2: Expression profiles of NRRL3_8837 and *lraA*; Figure S3: Result of SDS–PAGE (sodium dodecyl sulfate–polyacrylamide gel electrophoresis) of purified LraA; Table S1: List of genomic data sources used for BLASTP search; Table S2: Statistical analysis of the expression of the genes in this study.
