# Supplementary material for: L-Rhamnose Dehydrogenase LraA of Aspergillus niger Shows High Substrate Specificity Matching Its Expression Profile
Source: J Fungi (Basel). 2025 Apr 10;11(4):301. doi: 10.3390/jof11040301 (PMC12028159; doi:10.3390/jof11040301)
Supplement: Supplementary file 1 [file jof-11-00301-s001.zip › jof-3543430-supplementary.pdf]

Figure S1. Alignment of *A. niger* LraA and 8837

```
A. niger LraA   --MPLPQILVGKVAAITGGLTGIGRAIALEYLRHGAKVAVNHLGGSKEEP
A. niger 8837   MALPACNLLKGKTAAITGGTTGIGRAIALAFITQGCNVAVNHLGLPQDEV
                  :*   ::* **.****** ********* :: :*.:******* .::*

A. niger LraA   LIEAMRKDVSEIIGSNNDSEQSSSRFILVAGDVTQPETGREFVAKAVEAF
A. niger 8837   HRHSLLDEVK----SIQQGGIAAGKILEIPGDVTAPETSINLVKEAVSQW
                  .:: .:*.    * ::.  ::.::: :.**** ***. ::* :**. :

A. niger LraA   GRLDVFVSNAGVCKFEEFLEVDPPLLGHTINTNLCGAFWATQAAARQMAL
A. niger 8837   GKLDIFVANAGVFKQAEFLQIESSLLDHSIDVNIKGCFYSCQAAARQMV-
                *:**:**:**** *  ***:::..**.*:*:.*: *.*:: *******.

A. niger LraA   AQSPPGGSIIGISSISALVGGGQQTHYTPTKAGVLSLMQSCAVALGKYGI
A. niger 8837   -KQGHGGSIIGISSVSALVGGGLQTHYTPTKAAVLSMMQSMAISLGKDRI
                 :.  *********:******* *********.***:*** *::***  *

A. niger LraA   RCNALLPGTIRTQLNDEDMSDPVKRTYMEGRIPLGRLGQPPDLAGPAVFL
A. niger 8837   RCNALMPGTIATQLADHDMKNPTKKAALEARIPLGRIGNPDDMAGPAVFL
                *****:**** *** *.**.:*.*:: :*.******:*:* *:*******

A. niger LraA   A. niger 8837
```

```
                          ****:* **..: :*.***:* ***
ACEELSGYVTGAQILVDGGLFVNLQ
ACEEMSRYVNATGLLADGGMFSNLQ
```

Figure S2. Expression profiles of NRRL3_8837 and *lraA*

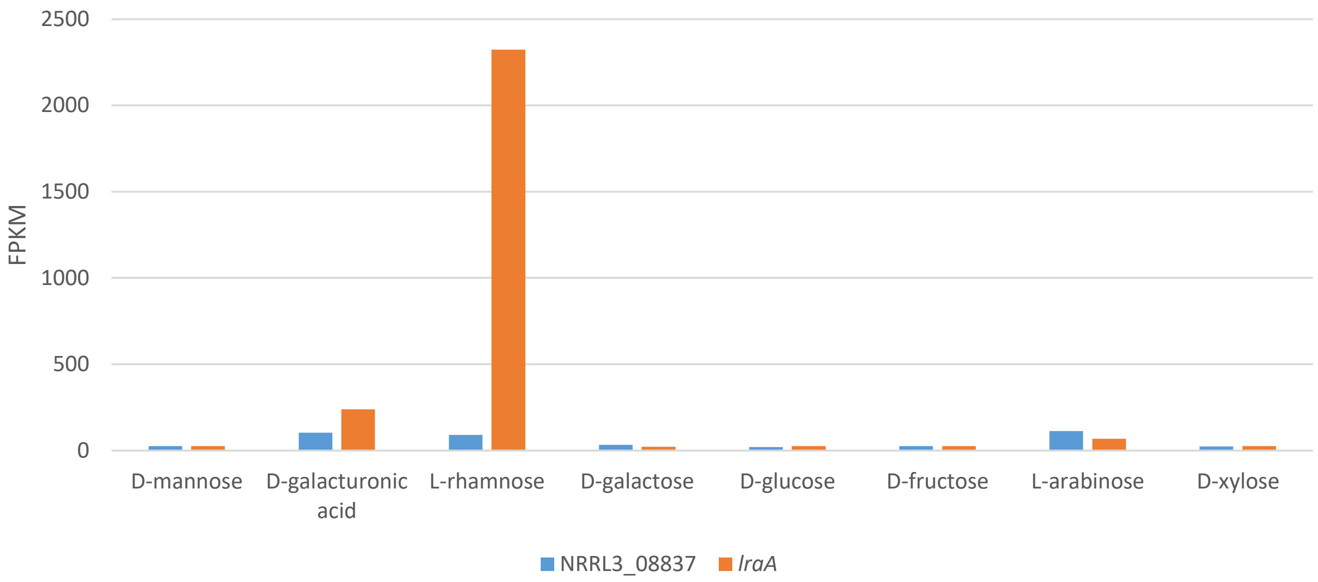

Figure S3. Result of SDS-PAGE (Sodium dodecyl sulfate-polyacrylamide gel electrophoresis) of purified LraA

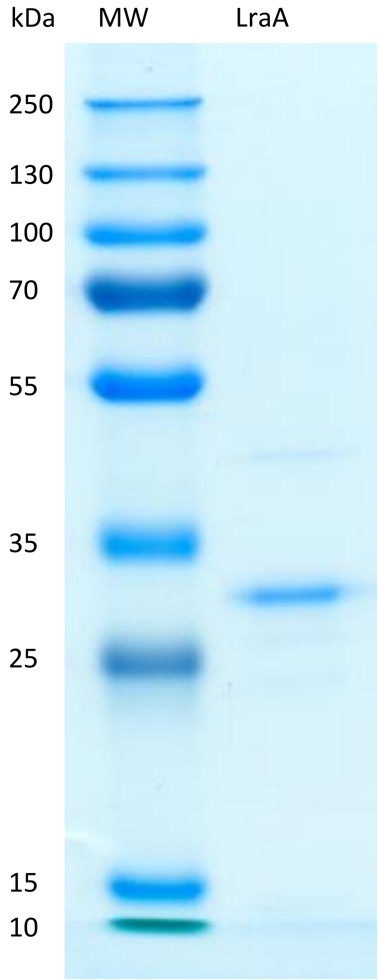

Table S1 List of genomic data sources used for BLAST P search.

Species	Gemone source
Aspergillus niger	https://mycocosm.jgi.doe.gov/Aspni_NRRL3_1/Aspni_NRRL3_1.home.html
Aspergillus nidulans	https://mycocosm.jgi.doe.gov/Aspnid1/Aspnid1.home.html
Chaetomium globosum	https://mycocosm.jgi.doe.gov/Chagl_1/Chagl_1.home.html
Neurospora crassa	https://mycocosm.jgi.doe.gov/Neucr2/Neucr2.home.html
Penicillium subrubescens	https://mycocosm.jgi.doe.gov/Pensub1/Pensub1.home.html
Podospora anserina	https://mycocosm.jgi.doe.gov/Podans1/Podans1.home.html
Trichoderma reesei	https://mycocosm.jgi.doe.gov/Trire_Chr/Trire_Chr.home.html

Supplemental Table S2. Statistical analysis of the expression of the genes of this study.

JGI protein ID	Gene	L.rhamnose_2h_VS_D.glucose_2h FoldChange	L.rhamnose_2h_VS_D.glucose_2h padj	L.rhamnose_2h_VS_D.fructose_2h FoldChange	L.rhamnose_2h_VS_D.fructose_2h padj	L.rhamnose_2h_VS_D.mannose_2h FoldChange	L.rhamnose_2h_VS_D.mannose_2h padj	L.rhamnose_2h_VS_D.galactose_2h FoldChange	L.rhamnose_2h_VS_D.galactose_2h padj	L.rhamnose_2h_VS_D.xylose_2h FoldChange	L.rhamnose_2h_VS_D.xylose_2h padj	L.rhamnose_2h_VS_L.arabinose_2h FoldChange	L.rhamnose_2h_VS_L.arabinose_2h padj	L.rhamnose_2h_VS_D.galacturonic.acid_2h FoldChange	L.rhamnose_2h_VS_D.galacturonic.acid_2h padj
jgi.p|Aspni_NRRL3_1|3278	RhtA	32540,2	6,64E-41	137301,9	3,12E-42	81302,1	1,46E-38	4251,1	2,14E-157	135252,5	3,19E-42	69191,0	1,33E-37	450,7	1,76E-114
jgi.p|Aspni_NRRL3_1|1494	lraA	1253,0	1,09E-89	1056,1	1,56E-105	1150,5	3,79E-109	414,8	8,24E-86	996,6	1,38E-104	154,0	1,79E-61	25,0	5,59E-21
jgi.p|Aspni_NRRL3_1|8837	homolog of lraA	59,7	3,14E-16	40,1	9,73E-17	64,2	2,14E-20	3,2	0,0	21,0	3,53E-12	4,5	0,001084762	0,1	1,97E-06
jgi.p|Aspni_NRRL3_1|1493	lrlA	7,3	5,73E-19	7,2	1,63E-22	9,0	9,42E-28	10,5	2,15E-31	7,4	3,58E-23	12,2	1,15E-35	6,0	4,75E-15
jgi.p|Aspni_NRRL3_1|1495	lrdA	2271,8	5,93E-88	1778,4	1,22E-100	3208,8	1,79E-114	774,3	6,88E-82	1795,3	5,12E-101	386,5	2,28E-66	44,2	2,55E-22
jgi.p|Aspni_NRRL3_1|8604	lkaA	112,3	4,75E-100	74,4	1,72E-101	69,8	4,66E-99	70,8	1,32E-99	62,1	1,00E-93	58,1	6,66E-91	1,2	0,69
jgi.p|Aspnid1|10128	RhtA	4,5	2,12E-17	5,9	2,11E-23	4,5	1,15E-17	7,5	3,82E-30	12,8	5,16E-45	12,8	1,66E-44	13,6	1,96E-45
jgi.p|Aspnid1|1902	lraA	113,2	3,94E-69	156,1	3,87E-78	86,3	8,81E-62	159,3	4,08E-79	218,5	3,67E-88	159,1	1,15E-78	89,4	1,41E-62
jgi.p|Aspnid1|7580	homolog of lraA	2,1	0,17	2,8	0,07	3,9	0,02	3,0	0,05	1,8	0,29	1,6	0,43	1,2	0,81
jgi.p|Aspnid1|2410	lrlA	74,7	5,26E-148	69,5	2,35E-143	64,8	6,16E-139	13,9	1,96E-58	30,8	4,77E-97	13,0	1,50E-55	25,2	5,27E-86
jgi.p|Aspnid1|10203	lrdA	275,6	1,55E-46	341,8	1,21E-49	259,4	1,27E-45	318,5	8,24E-49	335,0	1,22E-49	169,9	6,20E-39	61,1	1,42E-25
jgi.p|Aspnid1|6974	lkaA	130,4	3,86E-92	127,0	5,24E-91	145,0	6,63E-96	267,8	5,68E-119	127,1	3,08E-91	27,6	1,10E-43	38,1	1,45E-52
jgi.p|Pensub1|12232	RhtA	4,1	1,47E-15	2,3	1,56E-07	4,5	6,36E-19	0,4	8,39E-09	3,9	3,36E-14	0,3	7,94E-23	0,2	9,65E-38
jgi.p|Pensub1|9011	lraA	556,1	0	623,6	0	1273,2	0	627,9	0	3374,0	0	362,3	0	32,7	0
jgi.p|Pensub1|11772	lrlA	120,2	0	95,9	0	162,5	0	42,4	0	226,0	0	26,8	0	24,3	0
jgi.p|Pensub1|9010	lrdA	973,6	0	987,3	0	984,9	0	591,2	0	449,7	0	88,8	0	16,5	0
jgi.p|Pensub1|10375	lkaA	51,2	0	49,8	0	69,8	0	74,7	0	63,3	0	28,4	0	7,6	1,07E-164
jgi.p|Trire_Chr|112795	RhtA	822,5	8,40E-209	303,4	5,43E-177	890,3	2,54E-192	69,7	1,04E-110	68,8	2,23E-111	24,6	5,48E-64	18,9	2,83E-54
jgi.p|Trire_Chr|118190	lraA	71,9	2,77E-144	52,7	1,77E-127	76,1	6,80E-135	135,5	8,39E-160	148,3	4,83E-184	5,7	2,53E-31	15,1	6,82E-75
jgi.p|Trire_Chr|114943	lrlA	2,1	5,51E-09	1,0	0,96	1,8	2,12E-06	2,0	2,20E-08	2,7	2,49E-16	0,9	0,63	1,1	0,57
jgi.p|Trire_Chr|118189	lrdA	4889,7	0	4247,9	0	4066,0	0	849,9	0	660,8	0	12,3	1,20E-161	18,1	6,90E-216
jgi.p|Trire_Chr|109435	lkaA	2,4	2,60E-12	1,7	3,66E-05	1,9	3,65E-07	2,3	3,18E-11	4,0	6,04E-30	0,5	3,91E-07	0,1	1,83E-74
